# Gangrene of the Lungs Following Small-pox

**Published:** 1836

**Authors:** 


### Art. V. — Gangrene of the Lungs following Small-Pox.

Some time in the month of September last, we were called to visit Mr. M'C., a wagon-maker, who had been confined for several weeks with confluent small-pox. When we first saw him the scabs were dropping off, and he appeared to be recovering from the small-pox; but he complained of pain in the left side of the thorax, which had been very severe during his entire confinement. His pulse was wiry and frequent, although the action of the heart and vessels were quite feeble, and the radial artery appeared very small. He had also been affected, from the time that the eruption began to make its appearance, with a distressing cough. He informed me that his attendant had not bled him, although he had frequently urged him to do so, alledging that his pulse was always too feeble, notwithstanding the other inflammatory symptoms ran very high. His skin was mostly dry, and his tongue much coated in the centre with red and polished edges.

We bled him sixteen ounces, and directed antimonials with resinous and mercurial cathartics. The blood did not separate well; the crassamentum being more or less dissolved in the serum, and presenting a dark and tarry appearance towards the bottom of the vessel. The pain in the breast and cough continuing, a large blister was applied over the chest on the day following the venesection. He now began to cough up large quantities of dark colored blood, which continued, notwithstanding all our attempts to arrest it, until the patient died—a week after we first saw him.

On examining the body eight hours after death, we found the heart unusually small, though healthy in appearance, and the left lung reduced, by gangrene, to a dark pulpy mass, which emitted a strong and very offensive odor. Where the pleura pulmonalis was not firmly attached to the walls of the chest it was covered with a condensed layer of coagulable lymph, which had become completely organized with a great number of vessels, filled with blood, passing through it in every direction. Indeed, it was so perfectly formed that it could be separated like other membranes from its various attachments. A specimen of this curious secondary tissue may be seen in the museum of the Cincinnati College.

The right lung was mostly healthy, although it was separated from the sides of the chest by a mass of dark colored fluid resembling that found in the left side of the thorax. No tubercular secretion was observed in any portion of the pulmonary organs.

This case is interesting, so far as it shows the result of a defective plan of treatment in the early periods of the disease. Had a copious venesection been resorted to, at the proper period, the life of the patient would, in all probability, have been preserved. It would be curious to learn how far the small size of the heart may have been instrumental in producing a weak pulse, and thus deceiving the first attendant. It also afforded an excellent specimen of secondary membrane, so completely formed that the vessels might have been injected, for they could be seen passing through it in every direction, by the naked eye. Portions of the broken down lung, after being reduced to a fluid mass, also entered the circulation, otherwise it could not have made its way into the adjacent parts where it was found.

We were assisted in the *post mortem* examination by professors Rives and Gross, of the Cincinnati College.

